# Advanced units: quality measures in urgency and emergency care

**DOI:** 10.1590/S1679-45082014GS2894

**Published:** 2014

**Authors:** Dan Carai Maia Viola, Eduardo Cordioli, Carlos Henrique Sartorato Pedrotti, Mauro Iervolino, Antonio da Silva Bastos, Luis Roberto Natel de Almeida, Henrique Sutton de Sousa Neves, Claudio Luiz Lottenberg

**Affiliations:** 1 Hospital Israelita Albert Einstein, São Paulo, SP, Brazil.; 2 Universidade Federal de São Paulo, São Paulo, SP, Brazil.

**Keywords:** Quality indicators, health care, Primary health care, Emergency medical services, Triage

## Abstract

**Objective:**

To evaluate, through care indicators, the quality of services rendered to patients considered urgency and emergency cases at an advanced emergency care unit.

**Methods:**

We analyzed data from managerial reports of 64,891 medical visits performed in the Emergency Care Unit of the Ibirapuera Unit at Care during the period from June 1st, 2012 through May 31st, 2013. The proposed indicators for the assessment of care were rate of death in the emergency care unit; average length of stay of patients in the unit; rate of unplanned return visits; admission rate for patients screened as level 1 according to the Emergency Severity Index; rate of non-finalized medical consultations; rate of complaints; and door-to-electrocardiogram time.

**Results:**

The rate of death in the emergency care unit was zero. Five of the 22 patients classified as Emergency Severity Index 1 (22.7%) arrived presenting cardiac arrest. All were treated with cardiopulmonary resuscitation and reestablishment of vital functions. The average length of stay of patients in the unit was 3 hours, 33 minutes, and 7 seconds. The rate of unscheduled return visits at the emergency care unit of the Ibirapuera unit was 13.64%. Rate of complaints was 2.8/1,000 patients seen during the period

**Conclusion:**

The model of urgency and emergency care in advanced units provides an efficient and efficaious service to patients. Both critically ill patients and those considered less complex can receive proper treatment for their needs.

## INTRODUCTION

One of the greatest difficulties for the healthcare manager is to objectively assess the quality of care given. In the work routine, the care team is focused on the best care possible, but the perception of quality on the part of the patients and family members may be very different from the feeling of good services delivered by the healthcare professionals.

Berwick^(1)^ introduced the concept that it is possible to use measurements of quality from other industrial sectors in healthcare services.

Novaes and Paganini^(2)^ defined as quality criteria, data of the structure of services such as certifications of the teams, facilities, specifications of the materials used, and process indicators.

Donabedian^(3,4)^ was one of the first authors to try to define measurement indexes for medical care. He observed that simple measurement indexes could be related to highly complex situations (such as those that occur in surgical procedures) and are representative of the problem (validation of the indicators). One constant concern was defining which problems were identified in the care system and what the final objective was. The author suggested that if the manager did not know where he/she wanted to get, they would not get there. This theory perfectly demonstrates the methodology of indicators and goals used constantly at healthcare services.

In another study, Donabedian^(5)^ defined seven attributes as support pillars that define quality in healthcare, namely, efficacy, effectiveness, efficiency, resource optimization, acceptability, legitimacy, and equity.

According to the World Health Organization,^(2)^ in order to have quality in care, the presence of a few factors is necessary, such as a high degree of professional competence, efficiency in use of resources, minimization of risks, high degree of patient satisfaction, and favorable effect on health (appropriate outcome).

The concept of value is strongly connected to the concept of quality. From this perspective, it is important to discern the point of view of the one who evaluates – in this case, the patients and their family members. This evaluation of the care delivered is often more subjective than objective and may be related to personal and cultural values, subject to the influence of situations of stress and anxiety that the emergency department environment conveys to the individual.^(6-8)^

Within this perspective of difficulty and subjectivity in evaluating quality of healthcare systems, there is a clear concept in world literature that it is necessary to define adequate metric criteria for the evaluation, with reproducible and comparable indicators. Such measurements convey objectivity to data that could be lost in the subjectivity of personal evaluations, as to the feeling of quality in the service and care received.^(9,10)^

At the Emergency Care Units of *Hospital Israelita Albert Einstein* (HIAE), a triage system is used according to the Emergency Severity Index scale, known at these units an ESI, proposed by Gilboy et al. and Tanabe et al.^(11,12)^

By this scale, patients are classified and prioritized as per the degree of severity of the disease by means of an estimate of the number of secondary resources needed for their care. In this way, less seriously ill patients have the possibility of using fewer resources of the system, and are classified as ESI 5, while those most critically ill tend to require four or more resources of the system, and are classified as priority 1. Patients with a risk 1 classification need immediate medical care; those classified as 2 and 3 need care in up to 15 minutes; and those classified as risk 4 and 5 should be seen in up to 30 minutes.^(11-14)^

In the care model used in the advanced units, all the patients who need hospitalization are taken to HIAE – Morumbi by ambulance with a physician. The emergency cases receive initial care at the unit and are transferred as soon as they are stable enough for transport.

At the external units, the indicators in reference to institutional protocols (cerebrovascular accident, acute myocardial infarct (AMI), and sepsis*) *are also measured and managed.

## OBJECTIVE

The objective of the study was to evaluate, by means of care indicators, the quality of care given to the patients considered urgency and emergency cases at an Advanced Unit of Emergency Care.

## METHODS

The data from management reports of the 64,891 visits (encounters) at the Emergency Care Unit of the Ibirapuera Unit at HIAE during the period of one year, *i.e*., between June 1st, 2012 and May 31st, 2013, were analyzed. This period was chosen for analysis, since as of May 2012, the Ibirapuera Unit was inaugurated and has operated at new facilities, and in June, 2012, the emergency care sector started to use the Hospital Management System (SGH, acronym in Portuguese), an electronic medical records system, in its totality. The study did not involve data from patient medical records, so it was exempt from the need for approval by the Research Ethics Committee of HIAE.

The mean age of the patients who used the Ibirapuera Emergency Care was 26.46 years, whereas in the pediatric population (up to 16 years), the mean age was 5.24 years, and in the adult population (over 16 years), the mean age was 41.02 years. As to sex, 53.5% of the patients were female.

The Advanced Unit of Emergency Care is open for 2 hours a day with services in Internal Medicine, General Surgery, and Pediatrics. The emergency care also has orthopedic appointments, from 8:00 am to 10:00 pm, every day. The 64,891 clinical visits were distributed among Internal Medicine (28,020), General Surgery (3,215), Pediatrics (21,901), Orthopedics (8,580), and Nursing (2,482– dressings and/or medications).

At the Emergency Care Unit, the patients are selected according to the degree of severity of their complaint by means of the ESI.^(11-13)^ Table 1 shows the distribution of medical visits according to the ESI.

**Table 1:** Distribution of patients selected according to the Emergency Severity Index system during the period from June 1st, 2012 to May 31st, 2013

ESI	n (%)
1	22 (0.03)
2	574 (0.88)
3	37,248 (57.40)
4	22,985 (35.42)
5	1,026 (1.58)
Without classification	3,036 (4.68)

Total	64,891 (100.00)

Source: Hospital Management System – SGH (acronym in Portuguese) of *Hospital Israelita Albert Einstein. *ESI: Emergency Severity Index.

We defined as gauges of quality in care, some indicators that could represent efficacy, effectiveness, efficiency, resource optimization, acceptability, legitimacy, and equity, and that could be measured by means of operational data from one emergency care unit.

All the data were obtained from managerial reports of the SGH.

The indicators proposed for the evaluation of care were:

– Death rate in the emergency: number of patients who died divided by the number of patients seen;
– Mean length of stay at the unit: representing the mean, in minutes, of the time that the patients spent from the moment of arrival until hospital discharge;
– Rate of unscheduled return visits: returns within a period of up to 15 days with complaints similar to those of the first visit or complaints that represent complications of the underlying disease and/or treatment proposed (evaluated by comparison of the ICD at discharge);
– Rate of hospitalization of ESI 1 patients: number of hospitalizations of patients considered ESI 1 in triage divided by the absolute number of patients considered at triage as ESI 1;
– Rate of non-finalized medical treatments: represents the number of patients who abandoned treatment while in the waiting room (they had already gone through triage with the nursing staff), added to the number of patients who left after the beginning of medical treatment, divided by the total number of cases seen at the unit;
– Rate of complaints: represents the total number of complaints via the Customer Service (SAC, acronym in Portuguese) made by the patients or family members, divided by the total number of cases seen during that period;
– Door to electrocardiography: time spent from the patient’s arrival until the performance of the electrocardiogram on those patients with suspected AMI seen at the external unit, as per institutional protocol.

## RESULTS

Twenty-two cases were seen and the patients were classified in triage as ESI 1, *i.e.,* with immediate need for resuscitation. The rate of death at the emergency unit was zero.

Five of the 22 patients classified as ESI 1 (22.7%) arrived in a situation of cardiorespiratory arrest. All were submitted to cardiopulmonary resuscitation treatment with reestablishment of their vital signs.

**Figure 1:**
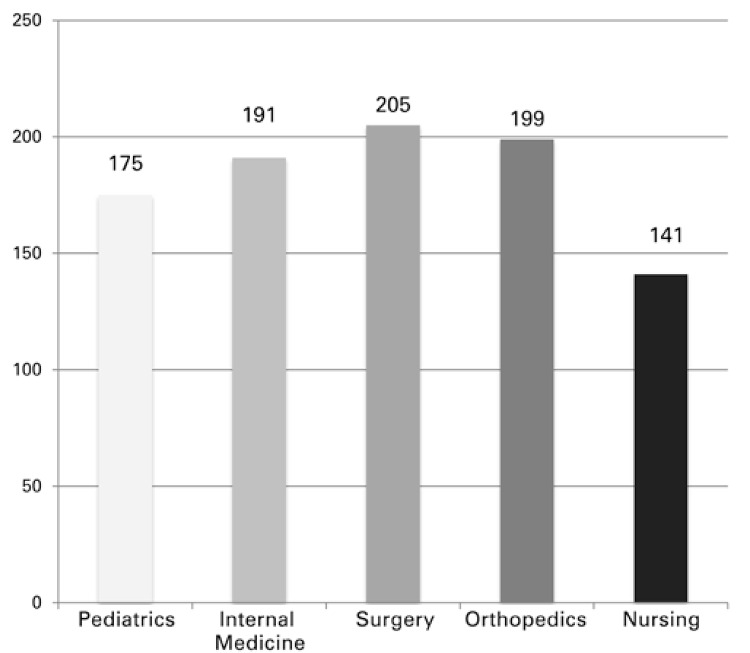
Mean length of stay of patients in the Emergency Care Unit (in minutes)

The mean length of stay of patients at the unit was 3 hours, 33 minutes, and 7 seconds (213 minutes) (Figure 1). Table 2 shows the mean length of stay of patients at the unit, as per the specialty and level of triage.

**Table 2:** Mean time in minutes, of patient stay when submitted to medical care, as per the specialty and level of triage

ESI	Pediatrics	Internal Medicine	General Surgery	Orthopedics	Mean
1	322	308	679	0	436.33
2	326	351	300	312	322.25
3	192	237	217	205	212.75
4	153	158	187	199	174.25
5	129	111	115	162	129.25

Hospital Management System – SGH (acronym in Portuguese) of *Hospital Israelita Albert Einstein. *ESI: Emergency Severity Index.

**Figure 2:**
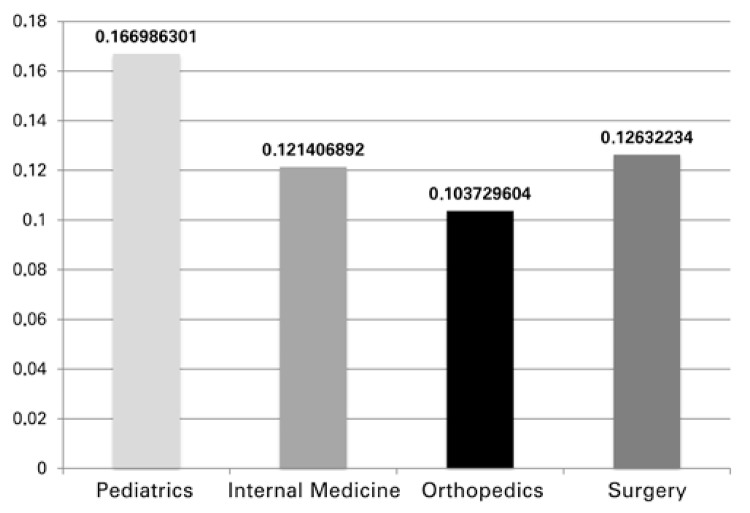
Rate of medical return visits within 15 days

The rate of unscheduled return medical visits was 13.64%. (Figure 2).

Of the 22 patients initially classified in triage as ESI 1, 13 were hospitalized (rate of hospitalization 59.1%). For the 64,891 patients seen, the rate of hospital admission was 1.2%.

**Figure 3:**
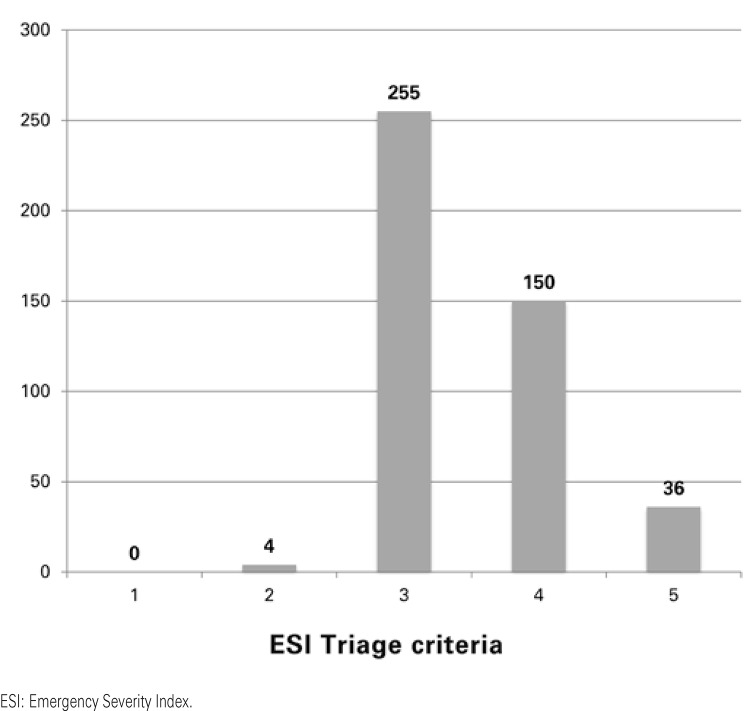
Distribution of the events at non-concluded medical visits, according to the level of triage of the Emergency Severity Index

Of the 64,891 patients classified in triage, 445 abandoned treatment before the end of the medical visit, which resulted in a rate of non-concluded medical visits of 0.7% (Figure 3).

A complaint rate of 2.8/1,000 cases seen (183 complaints) during the period analyzed. Of these, 53% were in reference to delays in being seen or to reevaluation.

The data from five patients diagnosed with AMI showing ST segment elevation were evaluated. The mean time for the electrocardiogram to be done was 7 minutes, and in 80% of the cases, the time was <10 minutes.

All patients with these diagnoses were referred to the hemodynamics sector of HIAE, transferred under emergency conditions in an ICU ambulance.

## DISCUSSION

The advanced unit model of emergency care aims to decentralize medical care. The basic proposal is to put closer the consumer market and the healthcare service, facilitating access of the population to emergency care. At first analysis, the primary focus would be to provide adequate medical care to patients with less complex health problems, which correspond to ESI triage system levels 3, 4, and 5. These patients generally use few resources from the institution and even more rarely need hospital admission. With this proposal, the model presented (the Advanced Unit of the Ibirapuera Emergency Care center) fulfills its basic function very well, since 94.4% of the patients seen are ESI 3, 4, and 5.

One important fact was the adaptation of the triage system to the patients seen. Analyzing the lengths of stay within the Emergency Care Unit, we observed that the patients of less complexity (ESI 4 and 5) remained shorter periods at the unit, when compared to those of greater complexity.^(14)^

When evaluating the absolute lengths of stay, the values seem very high for an Emergency Care Unit. It is important to note that the times are measured from the moment the patient gets the patient ticket number (before triage and registration) until the final release of the system (which often is done some time after the patient has been discharged from hospital).

The patient triage process, as per the ESI scale,^(12-14)^ defines five levels of complexity of care. No triage scale is infallible, and there may be incorrect allocations of patients. In our model, we observed 22 patients allocated as level 1 (red, requiring immediate resuscitation), but only 59.1% of these patients were hospitalized.

Excessive allocation of a level immediately more severe is the result of conservative safety mechanisms in patient care in order to prevent critically ill patients from not being timely diagnosed and treated. A second point is the learning curve in the use of the scale: this scale has been used for little over one year, and in spite of the team having been trained, it may take longer to get used to the criteria.

Decentralization of care plays a fundamental role in health service queues. Each year, at least 60 thousand patients did not seek Emergency Care at the Morumbi Unit of HIAE. On the other hand, decentralization expands the access of patients, since those who otherwise would not seek care at the organization (due to geographical location, for example), can now have a complete healthcare service near their homes.

In order to absorb all the demands of the population, the Advanced Units of Emergency Care need to have a minimal structure - clinical staff and facilities - that is sufficient to meet all demands. The division of care into Adult Internal Medicine, General Surgery, Pediatrics, and Orthopedics provides great power of resolution for the needs of the patients.

The patients that demanded specific care have at their disposal the possibility of calling a specialized physician for evaluation at the same advanced unit, and when necessary, there is the possibility of admission to the Morumbi Unit of HIAE.

The entire process of hospital admission is performed through the advanced unit itself. The patients are allocated to observation beds under the care of the medical and nursing teams while the bureaucratic procedures are taken care of. When the hospital bed is available, the patient is transported via ambulance to HIAE, accompanied by physicians and nursing professionals.

Despite the fact that most cases are related to patients of lesser complexity, the Advacend Unit of Emergency Care is prepared for urgency and emergency situations. At the time of triage, about 1% of the patients already present with life-threatening situations and need urgent or emergent medical care. If we consider the patients who progress with a worsening of risk, there are approximately one thousand patients a year with cases considered severe.

According to the organizational standards, the advanced units have equipment and trained professionals required to evaluate, identify, diagnose, and initiate treatment of all the patients in life threatening situations. The mortality rate for the model presented (zero in the last year) and the high rate of success in the resuscitation procedures in cardiorespiratory arrest demonstrate that the initial care, in situations of urgency and emergency, should and can be done at advanced units. In these situations, after the patient is stabilized, he/she is immediately transferred to the Morumbi Unit of HIAE to continue treatment.

The primary factor of success in emergency cases is the time elapsed until the start of adequate care, especially in AMI. In its most severe form, that is, AMI with supravanleveling ST segment, there is much evidence that delays in providing treatment with reperfusion of the obstructed coronary artery are related to increase in complications and early cardiovascular mortality.^(15)^

The HIAE, through its Cardiology Specialties Program, monitors each step of care of patients with chest pain. Clinical and epidemiological data as well as diagnostic procedures and medication administrations are documented, with their respective times, for the adjustment of quality goals. Thus, in addition to the “door-to-balloon” time, the period of each step of care is obtained.

At the advanced units, one of the key times is that period spent by the patient from arrival with the typical complaint until the electrocardiographic diagnosis (door-to-electrocardiogram time). In the model used for our study, the door-to-electrocardiogram time remained within standards considered ideal in treatment (less than 10 minutes), in cases of suspected AMI.^(15,16)^

In this situation, the advanced unit plays a fundamental role in the healthcare system, bringing the population closer to appropriate medical care. In a city with serious transportation problems such as São Paulo, the time spent until the patient arrives at a hospital may be a decisive factor in the result of treatment. In this way, emergency care at an advanced unit (decentralized from the hospital) may guarantee a greater chance of success in the outcome.

Despite bringing the hospital structure closer to the patients, the advanced unit is not always capable of satisfying all the desires and needs of the population. One frequent complaint over the last few months has been related to delay in care (53% of complaints). We observed that 0.7% of the patients did not complete their medical evaluation, and abandoned the clinical visit before medical care or after the initial tests were ordered (*i.e*., they did not await the results).

Shaikh et al.^(17)^ studied this phenomenon and found that 50% of the patients were willing to wait for up to 2 hours in an emergency care (before seeing the physician). Whereas Johnson et al.^(18)^ found a rate of 1.1% of patients who left the Emergency Care Unit early. The primary causes reported were waiting time for the medical visit and spontaneous resolution of the problem.

Such a situation occurred due especially to the increased demand of patients and the resulting waiting lines. At the unit used as model for this study, there has already been an adjustment in the clinical staff (physicians and nursing professionals) to balance the offer of services and the demand of the population.

Another demand from the population was the continuation of the treatment proposed. In addition to providing safety to the patient, the return visit to an appropriate environment guarantees maintenance of the entire productive chain of the healthcare system. In most cases, discharge from the emergency care does not finalize the treatment, and appropriate follow-up of the patient is needed. At the Ibirapuera Advanced Unit, the rate of return visits to the emergency care within 15 days was over 10%.

Some patients returned due to worsening of symptoms or changes in the clinical picture. However, many patients ended up using the emergency care as an outpatient center. This pattern of behavior is multifactoral. The identification of patients and of the causes of returns to the emergency care is fundamental for helping the patients go through the adequate follow-up, while at the same time, diminishing the demand for visits at the emergency care.

Recently, Hu et al.^(19)^ assessed the unexpected returns to emergency units. The authors observed a return rate of 3.1% (within 3 days) of cases and pointed out that the primary reasons for individuals returning were a more elderly population, patients classified at triage as seriously ill, and the presence of chronic diseases. On the other hand, Goldman et al.^(20)^ found an unscheduled return rate of 5% (within 72 hours) in the pediatric population.

The advanced unit works as an arm of the hospital, bringing the population to appropriate medical care. At the advanced unit, it is possible to separate the patients based on the degree of disease severity (triage); prioritize the care of the more seriously ill patients; diagnose and initiate treatment; safely transfer the patients who need hospitalization; and treat non-seriously ill patients with greater agility.

The major challenges in maintenance of quality of care are to scale and to maintain the trained medical and nursing team in order to meet the needs and demands of the population. The finalization of the healthcare system is possible with outpatient follow-up (medical specialties offices), closing the healthcare system cycle.

## CONCLUSION

The model of urgency and emergency care of advanced units affords an efficient and efficacious management. Both seriously ill patients and less complexity cases can receive adequate treatment for their needs.
