# Supplementary material for: Advanced units: quality measures in urgency and emergency care
Source: Einstein (Sao Paulo). 2014 Oct-Dec;12(4):492–8. doi: 10.1590/S1679-45082014GS2894 (PMC4879918; doi:10.1590/S1679-45082014GS2894)
Supplement: Translation

# Translation: Unidades avançadas: medidas de qualidade no atendimento de urgência e emergência

**Authors:** Dan Carai Maia Viola, Eduardo Cordioli, Carlos Henrique Sartorato Pedrotti, Mauro Iervolino, Antonio da Silva Bastos, Luis Roberto Natel de Almeida, Henrique Sutton de Sousa Neves, Claudio Luiz Lottenberg

**Affiliations:** 1 Hospital Israelita Albert Einstein, São Paulo, SP, Brasil.; 2 Universidade Federal de São Paulo, São Paulo, SP, Brasil.

**Keywords:** Indicadores de qualidade em assistência à saúde, Atenção primária à saúde, Serviços médicos de emergência, Triagem

## Abstract

**Objetivo:**

Avaliar, por meio de indicadores assistenciais, a qualidade do atendimento prestado aos pacientes considerados de urgência e emergência em uma Unidade Avançada de Pronto Atendimento.

**Métodos:**

Foram analisados os dados de relatórios gerenciais das 64.891 consultas (passagens) realizadas na Unidade de Pronto Atendimento da Unidade Ibirapuera do Hospital Israelita Albert Einstein no período de 1^o^ de junho de 2012 até 31 de maio de 2013. Os indicadores propostos para a avaliação do atendimento foram: taxa de óbito no pronto atendimento; tempo médio de permanência dos pacientes dentro da unidade; taxa de consulta de retorno não programado; taxa de internação dos pacientes com triagem 1 segundo o Índice de Severidade de Emergência; taxa de atendimento médico não finalizado; taxa de reclamações; e tempo porta-eletrocardiograma.

**Resultados:**

A taxa de óbito no pronto atendimento foi zero. Cinco dos 22 pacientes triados como 1 segundo o Índice de Severidade de Emergência (22,7%) chegaram em situação de parada cardiorrespiratória. Todos foram submetidos ao tratamento de reanimação cardiopulmonar com o reestabelecimento de suas funções vitais. O tempo médio de permanência dos pacientes dentro da unidade foi de 3 horas, 33 minutos e 7 segundos. A taxa de retornos em consulta médica não programados no Pronto Atendimento da Unidade Ibirapuera foi de 13,64%. Foi observada taxa de reclamações de 2,8/1.000 atendimentos realizados (183 reclamações) no período analisado.

**Conclusão:**

O modelo de atendimento de urgência e emergência de unidades avançadas propicia um atendimento eficiente e eficaz aos pacientes. Tanto os pacientes graves, quanto os de menor complexidade, podem receber o tratamento adequado as suas necessidades.

## INTRODUÇÃO

Uma das maiores dificuldades do gestor de saúde é aferir a qualidade do serviço prestado de forma objetiva. Na rotina de trabalho, a equipe assistencial está focada na melhor assistência possível, porém a percepção da qualidade, por parte dos pacientes e familiares, pode ser muito diferente da sensação de bom serviço prestado pelos profissionais da saúde.

Berwick^(1)^ introduziu o conceito de que é possível utilizar medidas de qualidade de outros setores industriais nos serviços de saúde.

Novaes e Paganini^(2)^ definiram como critérios para qualidade dados da estrutura dos serviços como certificações das equipes, instalações, especificações dos materiais utilizados e indicadores de processo.

Donabedian^(3,4)^ foi um dos primeiros autores que tentou definir índices de medida para o atendimento médico. Ele observou que índices de medida simples podem ser relacionados com situações altamente complexas (como as que ocorrem em procedimentos cirúrgicos) e, dessa forma, são representativos do problema (validação dos indicadores). Uma preocupação constante era definir quais os problemas encontrados no sistema de atendimento e qual o objetivo final. O autor preconizou que, se o gestor não sabe aonde quer chegar, não conseguirá chegar. Essa teoria demonstra perfeitamente a metodologia de indicadores e metas utilizadas constantemente nos serviços de saúde.

Em outro trabalho, Donabedian^(5)^ definiu sete atributos como pilares de sustentação que definem a qualidade em saúde, quais sejam: eficácia, efetividade, eficiência, otimização dos recursos, aceitabilidade, legitimidade e equidade.

Segundo a Organização Mundial da Saúde,^(2)^ para haver qualidade assistencial, é necessária a presença de alguns importantes fatores, como alto grau de competência profissional, eficiência na utilização dos recursos, minimização dos riscos, alto grau de satisfação dos pacientes e efeito favorável na saúde (desfecho apropriado).

O conceito de valor está fortemente conectado ao conceito de qualidade. Nessa perspectiva, é importante diferenciar o ponto de vista de quem avalia − no caso, os pacientes e os familiares. Essa avaliação do serviço prestado é, por vezes, muito mais subjetiva do que objetiva e pode estar relacionada a valores pessoais e culturais, sendo influenciados pela situação de estresse e de ansiedade que o atendimento de pronto-socorro traz ao indivíduo.^(6-8)^

Dentro dessa perspectiva de dificuldade e de subjetividade na avaliação de qualidade de sistemas de saúde, há um conceito claro na literatura mundial de que é necessário definir critérios métricos apropriados para avaliação, com indicadores reprodutíveis e comparáveis. Tais medidas trazem ao plano objetivo dados que podem se perder na subjetividade das avaliações pessoais, quanto a sensação de qualidade do serviço recebido.^(9,10)^

Nas Unidades de Pronto Atendimento do Hospital Israelita Albert Einstein (HIAE), é utilizado o sistema de triagem dos pacientes segundo a escala Índice de Severidade de Emergência, conhecida nessas unidades como ESI (*Emergency Severity Index*), proposta por Gilboy et al. e Tanabe et al.^(11,12)^

Nessa escala, os pacientes são classificados e priorizados conforme o grau de gravidade da doença, por meio da estimativa do número de recursos necessários para o atendimento. Dessa forma, temos que os pacientes menos graves têm a previsibilidade de utilizar menos recursos do sistema, sendo classificados como ESI 5, enquanto que os mais graves estão propensos a utilizar quatro ou mais recursos do sistema, sendo classificados como prioridade 1. Os pacientes com classificação de risco 1 necessitam de atendimento médico imediato; os classificados como 2 e 3 necessitam de atendimento em até 15 minutos; e os com classificação de risco 4 e 5 devem ser atendidos em até 30 minutos.^(11-14)^

No modelo de atendimento das unidades avançadas, todos os pacientes que necessitam de internação são encaminhados para o HIAE – Morumbi por de ambulância com médico. Os casos de emergência recebem o primeiro atendimento na unidade e são transferidos tão logo o paciente esteja estável para o transporte.

Nas unidades avançadas, os indicadores referentes aos protocolos institucionais (acidente vascular cerebral, infarto agudo do miocárdio (IAM) e sepse*) *também são medidos e gerenciados.

## OBJETIVO

O objetivo do estudo foi avaliar, por meio de indicadores assistenciais, a qualidade do atendimento prestado aos pacientes considerados de emergência e urgência em uma Unidade Avançada de Pronto Atendimento.

## MÉTODOS

Foram analisados os dados de relatórios gerenciais das 64.891 consultas (passagens) realizadas na Unidade de Pronto Atendimento da Unidade Ibirapuera do HIAE, no período de 1 ano compreendido entre 1º de junho de 2012 até 31 de maio de 2013. Foi escolhido esse período para análise, pois, desde maio de 2012, a Unidade Ibirapuera foi inaugurada e passou a operar em um novo espaço e, em junho de 2012, o setor do pronto atendimento passou a utilizar na íntegra o Sistema de Gestão Hospitalar (SGH), um sistema eletrônico de prontuário médico. O estudo não envolveu dados de prontuário dos pacientes, de forma que foi isento de aprovação por parte do Comitê de Ética em Pesquisa do HIAE.

A média de idade dos pacientes que utilizaram o Pronto Atendimento Ibirapuera no período foi de 26,46 anos, sendo que, na população pediátrica (até 16 anos), a idade média foi de 5,24 anos e, na população adulta (acima de 16 anos), a média de idade foi 41,02 anos. Quanto ao sexo, 53,5% dos pacientes eram do sexo feminino.

A Unidade Avançada de Pronto Atendimento funciona 2 horas por dia com atendimentos em clínica médica, Cirurgia Geral e Pediatria. O pronto atendimento apresenta ainda consultas de ortopedia no período das 8h às 22h, diariamente. As 64.891 consultas foram distribuídas entre Clínica Médica (28.020), Cirurgia Geral (3.215), Pediatria (21.901), Ortopedia (8.580) e Enfermagem (2.482 − curativos e ou medicações).

Na Unidade de Pronto Atendimento, os pacientes são triados conforme o grau de severidade de sua queixa por meio do sistema de escala ESI.^(11-13)^ Na tabela 1 observamos a distribuição das consultas conforme o ESI.

**Tabela 1:** Distribuição dos pacientes triados conforme o sistema Índice de Severidade na Emergência durante o período de 1o de junho de 2012 a 31 de maio de 2013

ESI	n (%)
1	22 (0,03)
2	574 (0,88)
3	37.248 (57,40)
4	22.985 (35,42)
5	1.026 (1,58)
Sem classificação	3.036 (4,68)
Total	64.891 (100,00)

Fonte: Sistema de Gestão Hospitalar do Hospital Israelita Albert Einstein. ESI: Índice de Severidade na Emergência.

Definimos como balizadores de qualidade do atendimento indicadores que pudessem representar eficácia, efetividade, eficiência, otimização dos recursos, aceitabilidade, legitimidade e equidade, e que pudessem ser medidos por meio de dados operacionais de uma unidade de pronto atendimento.

Todos os dados foram obtidos de relatórios gerenciais do SGH.

Os indicadores propostos para a avaliação do atendimento foram:

– Taxa de óbito no pronto atendimento: número de pacientes que evoluíram para óbito dividido pelo número de atendimentos realizados;
– Tempo médio de permanência na unidade: representa a média, em minutos, do tempo que os pacientes dispenderam desde o momento de sua chegada até a alta hospitalar;
– Taxa de consulta de retorno não programado: retornos em período de até 15 dias com queixas similares a do primeiro atendimento ou queixas que representem complicações da doença de base e/ou do tratamento proposto (avaliado pela comparação do CID de saída);
– Taxa de internação dos pacientes ESI 1: número de internações dos pacientes triados como ESI 1 dividido pelo número absoluto de pacientes triados como ESI 1;
– Taxa de atendimento médico não finalizado: representa o número de pacientes que desistiram do atendimento enquanto aguardavam na sala de espera (já haviam realizado a triagem de enfermagem) somado ao número de pacientes que se evadiram após o início do atendimento médico, dividido pelo número total de atendimentos na unidade;
– Taxa de reclamações: representa o número total de reclamações via Serviço de Atendimento ao Cliente (SAC) realizadas pelos pacientes ou familiares, dividido pelo número total de atendimentos realizados no período;
– Tempo porta-eletrocardiograma: é o tempo despendido desde a chegada do paciente até a execução do eletrocardiograma nos pacientes com suspeita de IAM atendidos na unidade avançada segundo o protocolo institucional.

## RESULTADOS

Foram realizados 22 atendimentos nos quais os pacientes foram triados como ESI 1, ou seja, com necessidade imediata de ressuscitação. A taxa de óbito no pronto atendimento foi zero.

Cinco dos 22 pacientes triados como ESI 1 (22,7%) chegaram em situação de parada cardiorrespiratória. Todos foram submetidos ao tratamento de reanimação cardiopulmonar com o reestabelecimento de suas funções vitais.

O tempo médio de permanência dos pacientes dentro da unidade foi de 3 horas, 33 minutos e 7 segundos (213 minutos) (Figura 1). Na tabela 2 observamos o tempo médio de permanência dos pacientes dentro da unidade, conforme a especialidade e o nível de triagem.

**Figura 1:** Tempo médio de permanência dos pacientes dentro da Unidade de Pronto Atendimento (em minutos)

**Tabela 2:** Tempo médio, em minutos, da permanência dos pacientes que foram submetidos ao atendimento médico, conforme a especialidade e o nível de triagem

ESI	Pediatria	Clínica Médica	Cirurgia Geral	Ortopedia	Média
1	322	308	679	0	436,33
2	326	351	300	312	322,25
3	192	237	217	205	212,75
4	153	158	187	199	174,25
5	129	111	115	162	129,25

Fonte: Sistema de Gestão Hospitalar do Hospital Israelita Albert Einstein.ESI: Índice de Severidade na Emergência.

A taxa de retornos em consulta médica, não programada, foi de 13,64%. (Figura 2).

**Figura 2:** Taxa de consultas médicas de retorno em até 15 dias

Dos 22 pacientes triados inicialmente como ESI 1, 13 foram internados (taxa de internação de 59,1%). Para os 64.891 pacientes atendidos, a taxa de internação foi de 1,2%.

Dos 64.891 pacientes triados, 445 desistiram do atendimento antes da finalização da consulta médica, perfazendo uma taxa de consultas não concluídas de 0,7% (Figura 3).

**Figura 3:** Distribuição dos eventos de consultas médicas não concluídas, conforme o nível de triage do Índice de Severidade na Emergência

Foi observada taxa de reclamações de 2,8/1.000 atendimentos realizados (183 reclamações) no período analisado. Destas, 53% foram referentes a demora no atendimento ou à reavaliação.

Foram avaliados os dados de cinco pacientes com diagnóstico de IAM com supradesnivelamento do segmento ST. O tempo médio para realização do eletrocardiograma foi de 7 minutos, com 80% dos casos com tempos <10 minutos.

Todos os pacientes com esses diagnósticos foram encaminhados para o setor de hemodinâmica do HIAE, sendo transferidos em condições de emergência na ambulância UTI.

## DISCUSSÃO

O modelo de Unidade Avançada de Pronto Atendimento visa descentralizar o atendimento médico. A proposta básica é aproximar o mercado consumidor do serviço de saúde, facilitando o acesso da população ao pronto atendimento. Em uma primeira análise, o foco principal seria proporcionar atendimento médico adequado aos pacientes com afecções de menor complexidade, os quais correspondem no sistema de triagem ESI aos níveis 3, 4 e 5. Esses pacientes geralmente utilizam poucos recursos da instituição e mais raramente necessitam de internação hospitalar. Com essa proposta, o modelo apresentado (a Unidade Avançada de Pronto Atendimento Ibirapuera) cumpre bem sua função básica, uma vez que 94,4% dos pacientes atendidos são ESI 3, 4 e 5.

Um outro dado importante foi a adequação do sistema de triagem aos pacientes atendidos. Analisando os tempos de permanência dentro da Unidade de Pronto Atendimento, observamos que os pacientes de menor complexidade (ESI 4 e 5) permanecem menos tempo dentro da unidade, quando comparados com os de maior complexidade.^(14)^

Avaliando os tempos absolutos, os valores parecem muito elevados para uma Unidade de Pronto Atendimento. Faz-se importante observar que os tempos são medidos desde o momento em que o paciente retira a senha (antes da triagem e da abertura de cadastro) até a alta final no sistema (que muitas vezes é realizada muito após a alta real do paciente).

O processo de triagem dos pacientes, segundo a escala ESI,^(12-14)^ define cinco níveis de complexidade de atendimento. Nenhuma escala de triagem é infalível, podendo haver alocações equivocadas dos pacientes. No nosso modelo, observamos 22 pacientes alocados como nível 1 (vermelhos, com necessidade de reanimação imediata), porém somente 59,1% desses pacientes foram internados.

A sobrealocação em nível imediatamente mais grave é o resultado de mecanismos conservadores de segurança do atendimento, para impedir que pacientes graves deixem de ser diagnosticados e tratados no momento oportuno. Um segundo ponto é a curva de aprendizagem no uso da escala: essa escala passou a ser utilizada há pouco mais de 1 ano e a equipe, apesar de treinada, pode demorar um tempo maior a se habituar com os critérios.

A descentralização do atendimento tem papel fundamental nas filas dos serviços de saúde. A cada ano, ao menos 60 mil pacientes deixaram de procurar o Pronto Atendimento da Unidade Morumbi do HIAE. Por outro lado, a descentralização expande o acesso, pois pacientes que outrora não buscariam o atendimento na instituição (pela localização geográfica, por exemplo) passam a ter serviço de saúde completo próximo de sua residência.

Para conseguir absorver todas as demandas da população, as Unidades Avançadas de Pronto Atendimento necessitam dispor de uma estrutura mínima (de corpo clínico e física) que seja suficiente para todas as demandas. A divisão do atendimento em Clínica Médica Adulta, Cirurgia Geral, Pediatria e Ortopedia traz grande poder de resolubilidade para as necessidades dos pacientes.

Os pacientes que demandam de cuidados específicos têm à sua disposição a possibilidade da convocação de um médico especialista para a avaliação na própria unidade avançada e, quando necessário, há a possibilidade de internação na Unidade Morumbi do HIAE.

Todo o processo de internação hospitalar é realizado dentro da própria unidade avançada. Os pacientes ficam alocados em leitos de observação enquanto ocorrem os trâmites burocráticos, sob os cuidados da equipe médica e de enfermagem. Quando o leito hospitalar está disponível, ocorre a transferência em ambulância para o HIAE, sendo o paciente acompanhado por médicos e profissionais de enfermagem.

Apesar da maioria dos atendimentos estar relacionada aos casos de menor complexidade, a Unidade Avançada de Pronto Atendimento está preparada para situações de urgência e emergência. Cerca de 1% dos pacientes já se apresentam, no momento da triagem, em situações de risco de morte e necessidade de atendimento médico de urgência e emergência. Se levarmos em consideração os pacientes que evoluem, durante o atendimento, com agravo de seu risco, temos aproximadamente mil pacientes ao ano com atendimentos considerados de pacientes graves.

Seguindo os padrões institucionais, as unidades avançadas dispõem de equipamentos e profissionais treinados para avaliar, identificar, diagnosticar e iniciar o tratamento de todos os pacientes em situações de risco de morte. A taxa de mortalidade no modelo apresentado (zero no último ano) e o alto índice de sucesso nos procedimentos de reanimação em situações de parada cardiorrespiratória demonstraram que o atendimento inicial, em situações de urgência e emergência, deve e pode ser realizado em unidades avançadas. Nessas situações, após a estabilização do paciente, o mesmo é imediatamente transferido para a Unidade Morumbi do HIAE para continuidade do tratamento.

O principal fator de sucesso em atendimentos de emergência é o tempo transcorrido até o início do atendimento adequado, especialmente no IAM. Em sua forma mais grave, ou seja, o IAM com supradesnivelamento de segmento ST, há ampla evidência de que atrasos na realização da terapêutica de reperfusão da coronária obstruída estão relacionados ao aumento de complicações e mortalidade cardiovascular precoce.^(15)^

O HIAE, por meio do Programa de Especialidades em Cardiologia, monitora cada etapa do atendimento de pacientes com dor torácica. São registrados dados clínicos e epidemiológicos, e anotados os procedimentos diagnósticos e administração de medicações, com respectivos horários, para ajuste de metas de qualidade. Assim, além do tempo porta-balão, obtém-se o tempo dispendido em cada etapa do atendimento.

Nas unidades avançadas, um dos tempos-chave é aquele dispendido pelo paciente da chegada na unidade com a queixa típica até a realização do diagnóstico eletrocardiográfico (tempo porta-eletrocardiograma). No modelo utilizado neste trabalho, o tempo porta-eletrocardiograma ficou dentro dos padrões considerados ideais no atendimento (menos de 10 minutos), nos casos de suspeita de IAM.^(15,16)^

Nessa situação, a unidade avançada cumpre papel fundamental no sistema de saúde, aproximando a população do atendimento médico adequado. Em uma cidade com sérios problemas de deslocamento, como São Paulo, o tempo gasto até o paciente chegar a um hospital pode ser decisivo no resultado do tratamento. Dessa forma, o pronto atendimento em unidade avançada (descentralizado do hospital) pode garantir maior chance de sucesso no desfecho do caso.

Apesar de trazer a estrutura hospitalar para próximo dos pacientes, nem sempre a unidade avançada consegue satisfazer todos os desejos e necessidades da população. Uma queixa frequente, nos últimos meses, foi quanto à demora no atendimento (53% das reclamações). Observamos que 0,7% dos pacientes não completaram sua avaliação médica, desistindo da consulta antes do atendimento médico ou após a solicitação de exames iniciais (não aguardaram os resultados).

Shaikh et al.^(17)^ estudaram esse fenômeno e encontraram que 50% dos pacientes estavam dispostos a esperar até 2 horas em um pronto atendimento (até o atendimento médico). Já Johnson et al.^(18)^ encontraram taxa de 1,1% de pacientes que deixaram precocemente a Unidade de Pronto Atendimento. As principais causas relatadas foram tempo de espera pela consulta e resolução espontânea do problema.

Tal situação ocorreu devido especialmente ao aumento de demanda de pacientes e as consequentes filas. Na unidade utilizada como modelo deste estudo, já houve uma adequação no corpo clínico (médicos e profissionais de enfermagem) para equilibrar a oferta de serviços e a demanda da população.

Outra demanda da população foi a perpetuação do tratamento proposto. Além de trazer segurança ao paciente, o retorno em ambiente apropriado garante a manutenção de toda cadeia produtiva do sistema de saúde. Na maior parte dos casos, a alta do pronto atendimento não finaliza o tratamento, sendo necessário um seguimento adequado do paciente. Na Unidade Avançada Ibirapuera, a taxa de retornos ao pronto atendimento em até 15 dias esteve acima de 10%.

Alguns pacientes retornam devido à piora dos sintomas ou por alterações no quadro clínico. Entretanto, muitos pacientes acabam utilizando o pronto atendimento como um centro ambulatorial. Esse padrão de comportamento é multifatorial. A identificação dos pacientes e das causas do retorno ao pronto atendimento é fundamental para auxiliar os pacientes a realizarem o seguimento adequado, ao mesmo tempo em que diminui a demanda por consultas no pronto atendimento.

Recentemente, Hu et al.^(19)^ avaliaram os retornos não esperados em unidades de emergência. Os autores observaram uma taxa de retorno de 3,1% (em até 3 dias) dos atendimentos e destacaram que as principais causas de retorno foram população mais idosa, pacientes triados como mais graves e presença de doenças crônicas. Já Goldman et al.^(20)^ encontraram taxa de retorno não programado de 5% (em até 72 horas) na população pediátrica.

A unidade avançada funciona como um braço do hospital, aproximando a população do atendimento médico adequado. Na unidade avançada é possível separar os pacientes segundo o grau de severidade da doença (triagem); priorizar os atendimentos dos pacientes mais graves; diagnosticar e iniciar o tratamento; transferir com segurança os pacientes que necessitam de internação; e tratar de forma ágil os pacientes menos graves.

Os principais desafios na manutenção da qualidade assistencial é dimensionar e manter treinada a equipe médica e de enfermagem para suprir as necessidades e as demandas da população. A finalização do sistema de saúde é possível com o seguimento ambulatorial (consultórios de especialidade), fechando o ciclo do sistema de saúde.

## CONCLUSÃO

O modelo de atendimento de urgência e emergência de unidades avançadas propicia um atendimento eficiente e eficaz. Tanto os pacientes graves, quanto os de menor complexidade, podem receber o tratamento adequado às suas necessidades.
